# Linear-Scaling Local Natural Orbital-Based Full Triples Treatment in Coupled-Cluster Theory

**DOI:** 10.1021/acs.jctc.4c01716

**Published:** 2025-02-21

**Authors:** Andy Jiang, Henry F. Schaefer, Justin M. Turney

**Affiliations:** Center for Computational Quantum Chemistry, Department of Chemistry, University of Georgia, Athens, Georgia 30602, United States

## Abstract

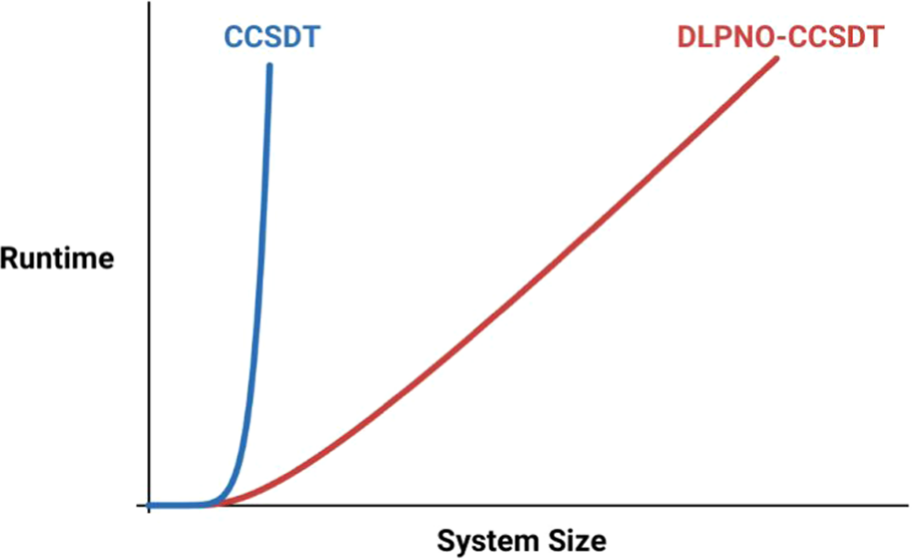

We present an efficient, asymptotically linear-scaling implementation of the canonically  coupled-cluster method with singles, doubles, and full triples excitations (CCSDT) method. We apply the domain-based local pair natural orbital (DLPNO) approach for computing CCSDT amplitudes. Our method, called DLPNO–CCSDT, uses the converged coupled-cluster amplitudes from a preceding DLPNO–CCSD(T) computation as a starting point for the solution of the CCSDT equations in the local natural orbital basis. To simplify the working equations, we *t*_1_-dress our two-electron integrals and Fock matrices, allowing our equations to take on the form of CCDT. With appropriate parameters, our method can recover more than 99.99% of the total canonical CCSDT correlation energy. In addition, we demonstrate that our method consistently yields sub-kJ mol^–1^ errors in relative energies when compared to canonical CCSDT, and, likewise, when computing the difference between CCSDT and CCSD(T). Finally, to highlight the low scaling of our algorithm, we present timings on linear alkanes (up to 30 carbons and 730 basis functions) and water clusters (up to 131 water molecules and 3144 basis functions).

## I Introduction

Coupled-cluster (CC) theory^1,2^ provides a systematic and size extensive means to solve the time-independent electronic Schrödinger equation
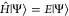
1

Full configuration interaction (FCI)^3,4^ provides the exact solution to the time-independent electronic Schrödinger equation within a finite basis set, by considering the interaction of every possible arrangement of electrons within a molecular orbital space. Unfortunately, FCI scales as  with respect to molecular and basis set size, making it intractable for molecules larger than a few atoms, even on the most advanced computer hardware. It is possible to reduce the number of configurations considered in CI methods by eliminating all excitations above a given order, though size extensivity issues limit the applicability of truncated CI. In contrast, coupled-cluster theory mitigates this issue by using an exponential ansatz.
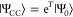
2
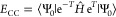
3

Ψ_0_ is obtained from a preceding Hartree–Fock computation. *T* represents the electron excitation operator in coupled-cluster theory, and *Ĥ* is the molecular Hamiltonian, within the Born–Oppenheimer approximation. With the electronic excitation operator, any number of electron excitations can be considered, up to every single active electron in the molecule. The more excitations considered, the closer the computed electronic energy should be to the FCI limit, at increasing computational time and resources. The cost of a coupled-cluster computation scales as  in time complexity, where *r* represents the max excitation order. For the  CCSDT method
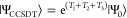
4
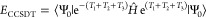
5

with *T*_1_, *T*_2_, and *T*_3_ being the one-, two-, and three-electron excitation operators, respectively. For coupled-cluster theory, relative energy errors of 1 kcal mol^–1^ or less are typically achievable only when triple excitations are considered.^5−8^ Historically, this required an exact treatment^9^ of triple excitations using CCSDT, a perturbative treatment using the CCSD(T) method,^10^ or through various simplifications of the full CCSDT method: CC*n*,^11−13^*n*CC,^14−16^ and CCSDT-*n* methods.^17−20^

CCSD(T) has become known as the “gold standard” method in quantum chemistry, due to its decent accuracy relative to its computational expense, and there have been a myriad of approximations devised to reduce the cost of the CCSD(T) method. These include but are not limited to density-fitting (DF)/resolution of the identity (RI),^21−23^ rank reduction (RR),^24−27^ tensor hypercontraction (THC),^26,28−32^ as well as localized orbitals.^33−54^ The latter include, in particular, the domain-based local pair natural orbital (DLPNO) approach of Neese and co-workers.^41,42,47,51,54^ Localized orbital approaches are also known as local correlation methods, and these methods are most popular due to its asymptotic linear scaling cost, and its tractability for larger molecules. With these local correlation methods, computations have been performed on a linear alkane containing 350 carbons in the def2-TZVPP basis (1252 atoms, 20678 basis functions) through the DLPNO–CCSD(T_0_)^41,42,47^ algorithm in ORCA,^55^ while the DLPNO–CCSD(T) algorithm,^51,54^ available in both ORCA and Psi4,^56^ has been performed on a whole insulin peptide in the def2-SV(P) basis (787 atoms, 6458 basis functions) through (T).^54^ These system sizes are much larger than what can be computed with canonical CCSD(T) on a typical lab workstation.

State-of-the-art local CCSD(T) methods include the domain-based local pair natural orbital [DLPNO–CCSD(T)] method^47,51,54^ in ORCA,^55^Psi4,^56^ and SERENITY;^57,58^ the pair natural orbital local [PNO-LCCSD(T)] method^48,52^ in Molpro;^59^ and the local natural orbital [LNO–CCSD(T)] method^50,53^ in MRCC.^60^ Through the proper selection of parameters,^43^ errors relative to canonical CCSD(T) are controllable, and errors relative to canonical CCSD(T) are typically proportional to system size, i.e., are “size extensive.”^61^

Though CCSD(T) offers a good balance between accuracy and cost for many systems, there are cases in which an exact treatment of triples would be helpful, such as when “chemical accuracy” (defined to be a 1 kcal mol^–1^) is not sufficient,^62−67^ or even required (e.g., molecules with a high multireference character).^68−73^ In addition, many focal point approaches utilize CCSDT in their extrapolations.^62,74,75^ Understandably, there has not been any significant interest in applying local correlation to post-CCSD(T) methods. Notable exceptions include the work of Schütz applying a projected atomic orbital (PAO) based approach for local CCSDT-1b (an approximation to CCSDT that uses e^*T*_1_+*T*_2_^ + *T*_3_ instead of e^*T*_1_+*T*_2_+*T*_3_^ as the excitation operator),^76^ and a pair natural orbital based approach to compute CC3 excitation energies of Frank et al.^77^ Nonlocal natural orbital approaches have been previously used with post-CCSD(T) methods, such as through the work of Rolik and Kallay^39^ in implementing CCSDT(Q)^78^ using MP2-based frozen natural orbitals (MP2 FNOs), although very tight FNO tolerances are often needed for sufficient accuracy.^79^ In contrast, localized natural orbitals offer both accuracy and efficiency even with tight natural orbital tolerances.^54^ With the success of local correlation methods at the CCSD(T) level of theory, there is a path to approach the FCI limit via the development of local natural orbital based methods for post-CCSD(T) methods such as CCSDT, CCSDT(Q),^78^ and CCSDTQ.^80−83^

To address this challenge, we have developed a local natural orbital-based implementation of the CCSDT method, following the methodology of the DLPNO methods of Neese et al.^41,42,47,51^ We derive our working equations from the work of Lesiuk,^84^ who presented working equations for CCSDT using *t*_1_-dressed integrals and Fock matrices, simplifying the equations to the complexity of CCDT. To highlight the accuracy and convergence of our code, we present computations on hydrogen cyanide and fulminic acid at a variety of triples natural orbital (TNO) tolerances.^85^ We also report reaction energies and barrier heights involving pericyclic reactions,^86^ showing sub-kJ mol^–1^ error relative to canonical CCSDT. In addition, we present timings on water clusters (up to 131 molecules), as well as linear alkanes (up to 30 carbons) to highlight the low-order scaling of our algorithm, with near-linear scaling achievable given sufficient sparsity, a drastic improvement from the  scaling of canonical CCSDT.

## II Theory

### II.I Notation

We use the following conventions to describe the indices of matrices and tensors appearing in this work, following our previous research^54^

- μ, ν, λ, σ: atomic orbitals; these range from 1 to *n*_bf_, the number of basis functions
- *i*, *j*, *k*, *l*: canonical and local occupied molecular orbitals; these range from 1 to *n*_occ_, the number of occupied orbitals
- *a*, *b*, *c*, *d*: canonical virtual molecular orbitals; these range from 1 to *n*_virt_, the number of virtual orbitals
- *p*, *q*, *r*, *s*: general canonical molecular orbitals; these range from 1 to *n*_occ_ + *n*_virt_
- μ̃, ν̃, λ̃, σ̃: projected atomic orbitals; these range from 1 to *n*_bf_
- μ̃_*ij*_, ν̃_*ij*_, λ̃_*ij*_, σ̃_*ij*_: projected atomic orbitals localized to pair *ij*; these range from 1 to *n*_pao,*ij*_, number of PAOs local to LMO pair *ij*
- μ̃_*ijk*_, ν̃_*ijk*_, λ̃_*ijk*_, σ̃_*ijk*_: projected atomic orbitals localized to triplet *ijk*; these range from 1 to *n*_pao,*ijk*_, number of PAOs local to LMO triplet *ijk*
- *a*_*ij*_, *b*_*ij*_, *c*_*ij*_, *d*_*ij*_: Pair natural orbitals in each pair domain *ij*; these range from 1 to *n*_pno, *ij*_, number of PNOs in the domain of LMO pair *ij*
- *l*_*ijk*_, *m*_*ijk*_, *n*_*ijk*_: “Interacting” local molecular orbitals in a triplet domain *ijk*, defined as the set of all occupied orbitals *l* such that *il*, *jl*, and *kl* are all strong or weak pairs; these range from 1 to *n*_lmo, *ijk*_, number of LMOs in the domain of LMO triplet *ijk*
- *a*_*ijk*_, *b*_*ijk*_, *c*_*ijk*_, *d*_*ijk*_: Triples natural orbitals in each triplet domain *ijk*; these range from 1 to *n*_tno, *ijk*_, number of TNOs in the domain of LMO triplet *ijk*
- *P*, *Q*: auxiliary basis functions for density-fitted ERIs; these range from 1 to *n*_aux_, number of auxiliary basis functions for density fitting
- *P*_*ij*_, *Q*_*ij*_: local auxiliary basis functions in each pair domain *ij*; these range from 1 to *n*_aux, *ij*_, number of auxiliary basis functions local to LMO pair *ij*
- *P*_*ijk*_, *Q*_*ijk*_: local auxiliary basis functions in each triplet domain *ijk*; these range from 1 to *n*_aux, *ijk*_, number of auxiliary basis functions local to LMO triplet *ijk*

The relative sizes of these indices are typically
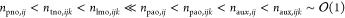
6
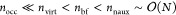
7where *N* is the system size represented by the number of atoms.

### II.II CCSDT Method and *t*_1_-Dressing

In the CCSDT method, the *T* cluster operator includes contributions from singles, doubles, and triples excitations. In the *t*_1_-dressed formalism, the effects of the *T*_1_ operator is folded into the Hamiltonian, analogous to its formulation with CCSD.^87^ We emphasize that the *t*_1_-dressing does not diminish the numerical accuracy of our results.
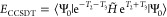
8where
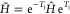
9
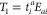
10
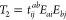
11
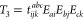
12

In restricted reference, closed-shell coupled-cluster theory, *E*_*ai*_ is defined as
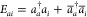
13where the barred creation/annihilation operators refer to the β spin orbitals and the nonbarred refer to the α spin orbitals. The quantity *t*_*i*_^*a*^ is known as the singles, *t*_*ij*_^*ab*^ the doubles, and *t*_*ijk*_^*abc*^ the triples amplitudes. The amplitudes are iterated to self-consistency through the solution of the following equations
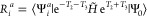
14
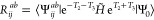
15
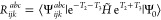
16with
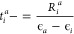
17
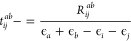
18
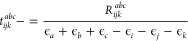
19where ϵ_*i*_ and ϵ_*a*_ represent orbital energies obtained from the diagonal elements of the Fock operator in the MO basis.

The residual update equations are much more simplified compared to the traditional formulation of CCSDT,^9^ since all terms involving singles excitations no longer arise explicitly. However, the *t*_1_-dressed formalism of coupled-cluster theory initially lacked popularity due to the cost of transforming four-center, two-electron integrals every CC iteration. However, DePrince et al.^21^ have shown that when the density-fitting (DF)/resolution of the identity^88−96^ or Cholesky decomposition (CD)^97−99^ approximations of two-electron integrals are applied, the use of the *t*_1_-transformed formalism becomes worthwhile. DePrince used *t*_1_-dressed integrals in his implementation of DF-CCSD,^21^ while Schwilk et al.^48^ and Jiang et al.^54^ used the *t*_1_-transformed formalism in their implementations of local pair natural orbital based CCSD. In this formalism, the two-electron integrals are approximated as
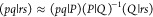
20where *P* and *Q* represent auxiliary basis functions. This can be rewritten as
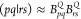
21where
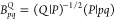
22The *t*_1_-dressed DF integrals can be presented as^21,48,54^
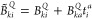
23
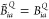
24
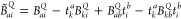
25
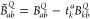
26while the *t*_1_-dressed Fock matrices are^48,54^
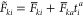
27
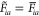
28
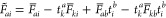
29
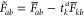
30where
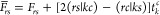
31

We present our working equations based on the prior work of Lesiuk.^84^ The singles residual expression is
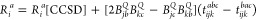
32

The doubles residual expression is
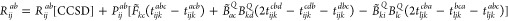
33*P*_*ij*_^*ab*^ is a permutation operator, and is defined as
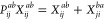
34

Analogously, we present the “long” and “short” permutation operators as, respectively^84^
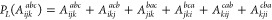
35
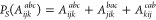
36

For the triples residual, we note a sign error from the original work of Lesiuk,^84^ and have adjusted the equations accordingly (the signs of every term in eq 11 from ref (84))
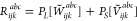
37

A tilde is added to the long and short permutation intermediates to distinguish them from similarly defined quantities in CCSD(T).^32,54^ These quantities are defined as
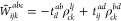
38where
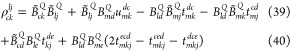
40
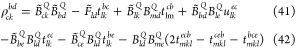
42*u*_*ij*_^*ab*^ = 2*t*_*ij*_^*ab*^ – *t*_*ij*_^*ba*^ represents the antisymmetrized doubles amplitude. For the short permutation intermediates
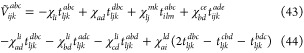
44where
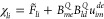
45
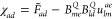
46
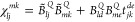
47
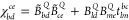
48
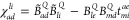
49
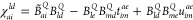
50

The energy expression is computed from the converged *t*_*i*_^*a*^ and *t*_*ij*_^*ab*^ amplitudes
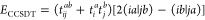
51

### II.III Overview of Domain-Based Local Pair Natural Orbital (DLPNO)

Since our DLPNO–CCSDT algorithm begins with our implementation^54^ of the DLPNO–CCSD(T) algorithm of Neese et al.,^36,41,42,47^ it is helpful to provide a brief overview of the algorithm for reader comprehension.

#### II.III.I Local Molecular Orbitals (LMOs)

After the completion of a Hartree–Fock procedure, one can apply a unitary transformation to the active occupied orbitals to limit their spatial extent^100^
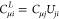
52

The Foster–Boys^101,102^ or Pipek–Mezey^102,103^ methods can be used to compute the unitary transformation matrix *U*. In this work, we use the Foster–Boys approach for all of our computations, following the work of Riplinger et al.^47^ and our previous work.^54^ We call the transformed set of active occupied molecular orbitals LMOs, or local molecular orbitals. In the DLPNO–CCSD algorithm,^47,54^ pairs of LMOs are classified as “dipole pairs,” “semi-canonical MP2 pairs,” “weak pairs,” and “strong pairs”; these pairs are divided based on a series of prescreening steps. The reader is referred to the work of Valeev, Neese, and co-workers^47^ and our previous work^54^ for a more comprehensive overview of the pair prescreening procedures. In the DLPNO-(T) algorithm, triplets (*i*,*j*,*k*) are determined from strong and weak pairs, so the number of relevant triplets also shows linear scaling. For more details on the triplet prescreening procedure performed in DLPNO-(T), the reader is referred to our previous work on DLPNO–CCSD(T).^54^

#### II.III.II Projected Atomic Orbitals (PAOs)

One of the earliest attempts at virtual space localization was through projected atomic orbitals (PAOs).^104^ Since the span of the atomic orbital (AO) space is the same as the molecular orbital (MO) space, a set of linearly dependent functions that span the virtual space can be formed by subtracting out the span of the occupied space from the complete AO space. These are known as projected atomic orbitals (PAOs). PAOs have a more local character compared to canonical virtual molecular orbitals.
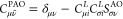
53
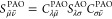
54

The *C*^PAO^ coefficients give the contribution of atomic orbital μ to PAO ν̃, and *S*^PAO^ represents the overlap matrix between two PAOs. Next, the PAOs are orthonormalized
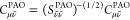
55and the PAO overlaps are noniteratively recomputed using the new PAO coefficients.

In earlier local correlation methods,^76,105−107^ PAOs are assigned to each LMO based on sparsity. Since there is a constant number of significant PAOs per LMO, these methods approach linear-scaling. PAO-based local correlation methods represent the first generation of linear-scaling post-Hartree–Fock methods, and are forerunners to pair natural orbital (PNO) based approaches.^36,37^ In DLPNO-based algorithms, assigning PAOs to each LMO is done as a precursor before PNOs are computed, to ensure the linear-scaling of PNO generation. In our work, following Pinski et al.,^46^ PAOs are assigned to each LMO based on spatial overlap, computed through a measure called the “differential overlap integral” (DOI)
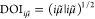
56

If the computed value of the DOI is greater than a given tolerance, parametrized as *T*_CUT_DO_ (default 0.01), then the PAO μ̃ is included in the domain of LMO *i*. For the pair domain of *ij* (denoted μ̃_*ij*_), the PAOs of domain *i* (μ̃_*i*_) are merged with the PAOs of domain *j* (μ̃_*j*_) to form the larger pair domain. Analogously, for triplet *ijk*, the PAOs of LMOs *i*, *j*, and *k* are merged to form the triples domain. Unfortunately, for PAO-based local correlation methods, there is a high crossover with these methods and their canonical counterparts, as the PAOs do not form the most sparse representation of the virtual space.

#### II.III.III Pair and Triples Natural Orbitals (PNOs and TNOs)

PNOs, initially formulated in the early days of quantum chemistry by Edmiston and Krauss as “pseudonatural orbitals,”^108^ saw initial popularity in the PNO–CI^109−111^ and the IEPA-PNO^112^ approaches. However, the method dwindled in use due to the rise in popularity of direct configuration interaction (direct CI) approaches.^113,114^

With the rise of local correlation methods, Neese et al. realized that PNOs can form a natural basis to express the virtual space of a given LMO pair, and developed the LPNO-CEPA^36^ and LPNO–CCSD^37^ methods. Since truncated PNOs represent the most compact representation of the virtual space of an LMO pair, PNO-based local correlation methods are orders of magnitude more efficient than their PAO counterparts. In doing so, they introduced the next generation of local correlation methods by resurrecting PNOs from the annals of quantum chemistry. PNOs are defined as the eigenvectors of the pair density of a molecular orbital pair *ij*.
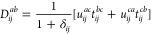
57

The amplitudes used in the pair density can be represented in any virtual molecular orbital space. Initially, Neese et al. used canonical MP2^115^ amplitudes to form the pair density.^36,37^ Later on, they updated their method by first assigning PAOs to each pair domain, and using *semicanonical* MP2 amplitudes for each pair in the PAO basis.^41^ This union of the early PAO and PNO approaches is the basis behind the domain-based local pair natural orbital (DLPNO) family of algorithms.^41,42,46,47,51,54^ In the DLPNO, the pair density is first diagonalized in the PAO basis of pair *ij*
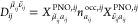
58where the eigenvectors, *X*_μ̃_*ij*_*a*_*ij*__^PNO,*ij*^, form the transformation matrix from PAOs to PNOs, and the eigenvalues *n*_*a*_*ij*__^occ,*ij*^ are the natural orbital occupation numbers.

The PNOs are then truncated by occupation number^41^ (*T*_CUT_PNO_, defaulting to 3.33 × 10^–7^) to form a compact virtual space for pair *ij*. In addition, energy^48,54^ and trace criteria^54^ can be used. After the truncated PNO basis is constructed, the truncated PNOs are canonicalized to give orbital energies for the pair *ij*. For singles, the virtual space is assigned using PNOs from diagonal pair ii, using a tighter occupation cutoff (*T*_CUT_PNO_ scaled by *T*_DIAG_SCALE_, with the scaling defaulting to 0.03). For triples, the triplet density^42^ is formed as the average of pair densities *ij*, *jk*, and *ik* formed from converged LCCSD doubles amplitudes (converged LMP2, LCEPA0, or LCCD amplitudes for weak pairs), and triples natural orbitals (TNOs) are formed from the eigenvectors of the triples density.
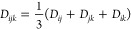
59

Currently, only the occupation criterion *T*_CUT_TNO_ (default 10^–9^) is used for the selection of TNOs. The use of analogous energy or trace criteria has not been considered but could be a worthwhile future investigation, especially in the context of exact triples.

#### II.III.IV Local Density Fitting

The effective use of the *t*_1_-transformed formalism requires the use of density-fitting (DF) for the computation of two-electron integrals. However, using the full set of auxiliary basis functions would remove the linear-scaling properties of the algorithm. Fortunately, since all of the integrals computed in DLPNO–CCSD/(T)/T are expressed using localized occupied or virtual subspaces, only a subset of the auxiliary basis functions is required for each pair domain. Following the work of Riplinger et al.,^41,47^ we first determine the Mulliken population of electrons of LMO *i* on each atomic center *A*(116)
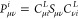
60
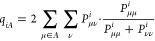
61If *q*_*iA*_ exceeds a tolerance (*T*_CUT_MKN_), defaulting to 10^–3^ for CCSD and 10^–2^ for post-CCSD computations, then all the fitting functions centered on atom *A* are assigned to the DF domain of LMO *i*. The local DF domain of pair *ij* is formed from the union of local fitting functions from LMOs *i*, and *j*, while the local DF domain of triplet *ijk* is formed from the union of fitting functions from LMOs *i*, *j*, and *k*.

#### II.III.V DLPNO-(T) Algorithm Additional Details

Since the initial guess triples amplitudes, the local density fitting (LDF), and the TNO domains for each triplet *ijk* for our DLPNO–CCSDT algorithm are formed from our previously implemented DLPNO–CCSD(T) algorithm (described in more detail in ref (54)), we deem it appropriate to give a very brief overview of the procedures and parameters used in our DLPNO-(T) algorithm. The DLPNO–CCSD(T) algorithm in our code combines features from the originally inspired DLPNO–CCSD(T) algorithm in ORCA^42,51^ as well as the PNO-LCCSD(T) algorithm in Molpro.^52^ First, we first compute a list of every possible triplet *ijk*, derived from combinations of pairs *ij*, *jk*, and *ik*, at least one of which is a strong pair. For efficiency in time and storage, we make use of restricted indexing *i* ≤ *j* ≤ *k*. The semicanonincal (T0) energy is computed for each possible triplet at a looser TNO tolerance (*T*_CUT_TNO_PRE_). All triplets *ijk* with energy contributions lower than (*T*_CUT_TRIPLES_PRE_) are not further considered, but their energy contributions will be accounted for. The nonsurviving triplets are deemed “screened triplets.” Next, the TNOs of each surviving triplet are recomputed at a tighter tolerance (*T*_CUT_TNO_), to form the initial semicanonical (T0) energy.
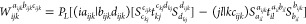
62
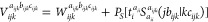
63
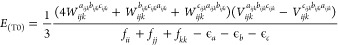
64

Next, to reduce the memory costs of needing to store triples amplitudes, the triplets are then subdivided into “strong triplets” and “weak triplets.” To this end, the energies of the triplets are sorted, and the triplets with the highest energies, that account for at least 90% of the semicanonical (T0) energy, are “strong triplets,” and the rest “weak triplets.” Typically, the set of strong triplets is around 20% of all the surviving triplets from prescreening. The TNOs are recomputed at tolerance *T*_CUT_TNO_ × *T*_STRONG_SCALE_ for strong triplets and the “weak triplets” and *T*_CUT_TNO_ × *T*_WEAK_SCALE_ for weak triplets. Next, the triples amplitude is solved self-consistently. This procedure is derived from the work of Guo et al.^51^
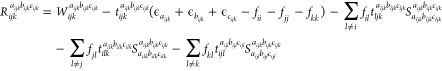
65
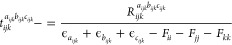
66
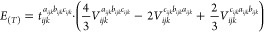
67

The final DLPNO-(T) energy expression is presented below after the triples amplitudes are converged. As a starting point for the DLPNO–CCSDT computation, the converged DLPNO–CCSD(T) triples amplitude, and DLPNO–CCSD singles and doubles amplitudes are used.
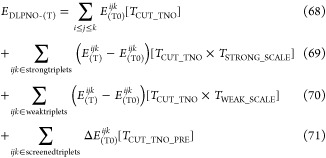
71

In Table 1, we present the default values of all triples related parameters. These parameters affect both the DLPNO–CCSD(T) and DLPNO–CCSDT algorithms.

**Table 1:** Default Values of Our DLPNO-(T) Parameters

parameter	description	default value
*T*_CUT_TNO_	TNO occupation criterion	10^–9^
*T*_CUT_TNO_PRE_	TNO occupation criterion in “screened triplets”	10^–7^
*T*_CUT_TRIPLES_PRE_	“Screened triplets” energy cutoff	10^–7^
*T*_CUT_DO_TRIPLES_	LMO/PAO DOI criterion for triples domains	10^–2^
*T*_CUT_MKN_TRIPLES_	local density fitting tolerance for triples	10^–2^
*T*_STRONG_tSCALE_	iterative (T) strong triplet *T*_CUT_TNO_ scaling	10
*T*_WEAK_SCALE_	iterative (T) weak triplet *T*_CUT_TNO_ scaling	100

## III DLPNO–CCSDT Algorithm

In this section, our DLPNO–CCSDT algorithm is presented. We have adapted our triples amplitudes to be index-restricted by *i* ≤ *j* ≤ *k*, to reduce memory requirements, so our algorithm and working equations have been adjusted accordingly. Our algorithm is asymptotically linear-scaling, due to the linear number of significant *ijk* triplets with increasing system size, and each triplet domain having an asymptotically constant number of interacting LMOs (*l*_*ijk*_), local auxiliary functions (*Q*_*ijk*_), as well as virtual orbitals (*a*_*ijk*_). For clarity, in our presented pseudocode, the interacting LMOs of triplet *ijk* are denoted “lmotriplet_to_lmos[*ijk*]”, and the set of all index-restricted triplets is denoted “lmo_triplets”. Every contraction in our algorithm takes advantage of the efficient level 2 matrix-vector (GEMV) or level 3 matrix–matrix (GEMM) BLAS operations, and these are denoted in the comments. In our algorithms, we denote projected amplitudes with a bar.
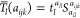
72
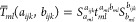
73
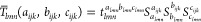
74We sometimes require the dense storage of projected singles and doubles amplitudes within a triplet domain for the sake of efficiency
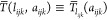
75
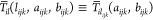
76
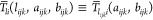
77
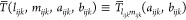
78

### III.I *R*_*i*_^*a*^ and *R*_*ij*_^*ab*^ Algorithms

Algorithm 1 and Algorithm 2 show how to compute *R*_*i*_^*a*^ and *R*_*ij*_^*ab*^, respectively. The permutation adapted working equations for computing the triples contributions to the *R*_*i*_^*a*^, and *R*_*ij*_^*ab*^ residuals have already been derived from the work of Paul et al.^117^ We present their equations adapted to the DLPNO basis below. First, we present the *u*_*ijk*_^*abc*^ intermediate, defined as τ̃_*ijk*_^*abc*^ in the work of Paul et al.^117^
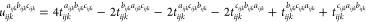
79The triples contribution to the singles amplitude takes the form
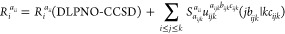
80

We define the permutation operators on a triples intermediate *X*(*a*_*ijk*_, *b*_*ijk*_, *c*_*ijk*_) as follows
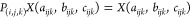
81
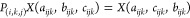
82
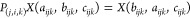
83
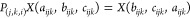
84
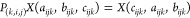
85
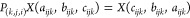
86
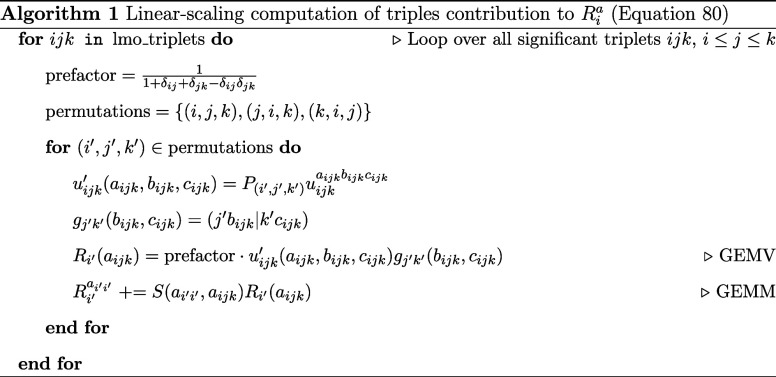


In our notation, (*i′*, *j′*, *k′*) represents a permutation of (*i*, *j*, *k*). For example, if (*i*′, *j*′, *k*′) = (*k*, *i*, *j*), then *i*′ = *k*, *j*′ = *i*, *k*′ = *j*. Similarly, permutational symmetry can be exploited for the contributions to *R*_*ij*_^*ab*^.^117^ Terms with a tilde represent *t*_1_-dressed quantities^54^
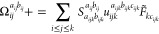
87
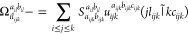
88
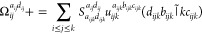
89
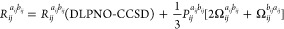
90

### III.II *R*_*ijk*_^*abc*^ Algorithm

Algorithms 3 and 4 depict how to compute the long and short permutation contributions to *R*_*ijk*_^*abc*^, respectively. The computation of the *R*_*ijk*_^*abc*^ residual has been adapted from the work of Lesiuk^84^. Using the conjugate permutation operator, from the permutation operator defined earlier (eqs 81–86)
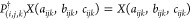
91
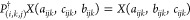
92
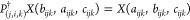
93
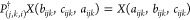
94
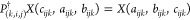
95
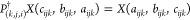
96
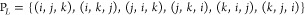
97
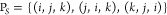
98
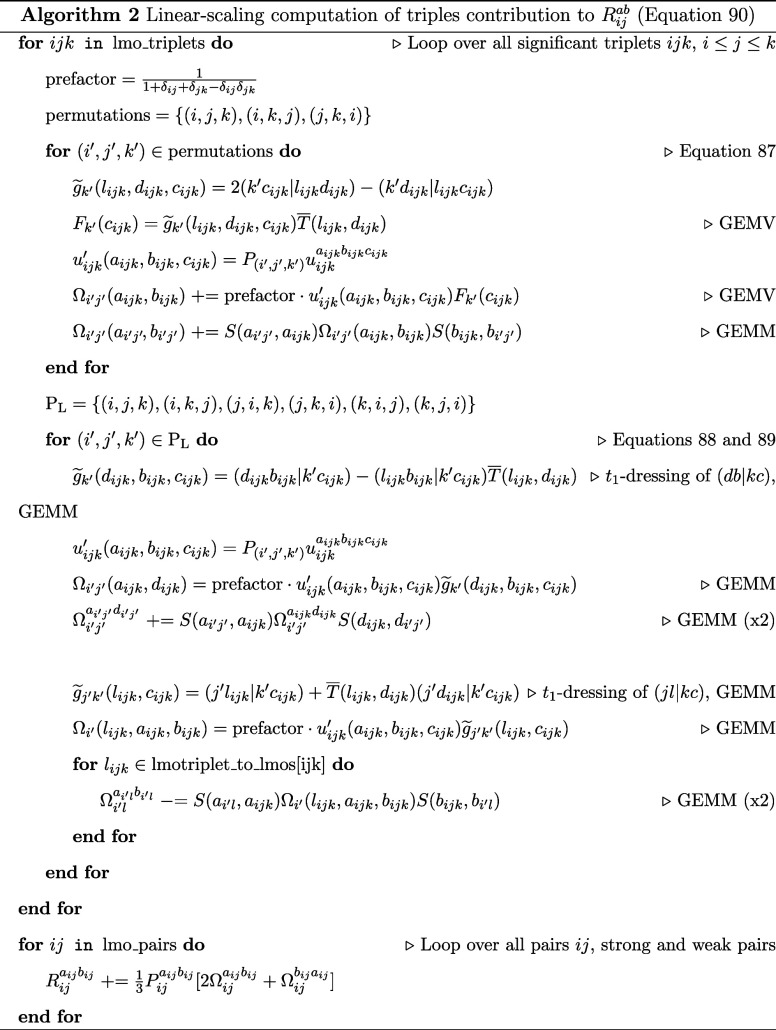


the permutation adapted triples residual equation in the TNO basis can be written as
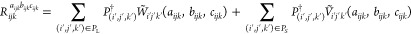
99where
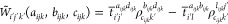
100We define
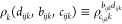
101
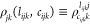
102
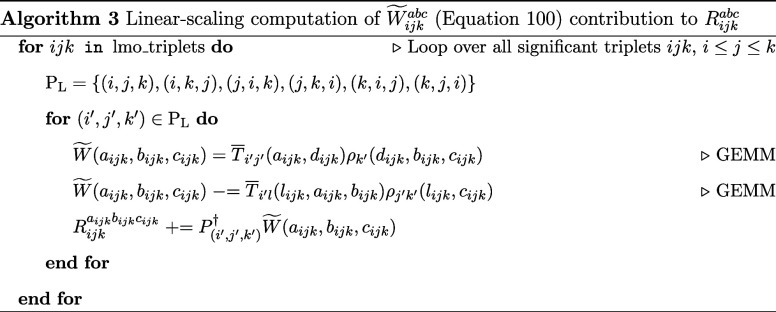


The algorithms for computing each of the ρ intermediates are presented in the Supporting Information. Similarly, for the*Ṽ* intermediate
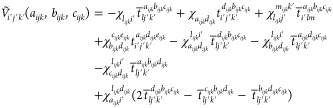
103

with the following terms in the algorithm below defined
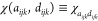
104
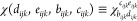
105
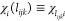
106
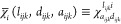
107
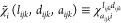
108
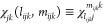
109
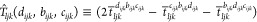
110

The algorithms for computing each of the χ intermediates are also presented in the Supporting Information.

### III.III Energy Contributions

The DLPNO–CCSDT energy can be expressed as the sum of the following contributions, from converged CCSDT singles and doubles amplitudes.
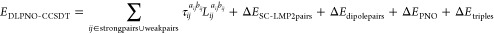
111with
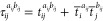
112
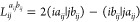
113
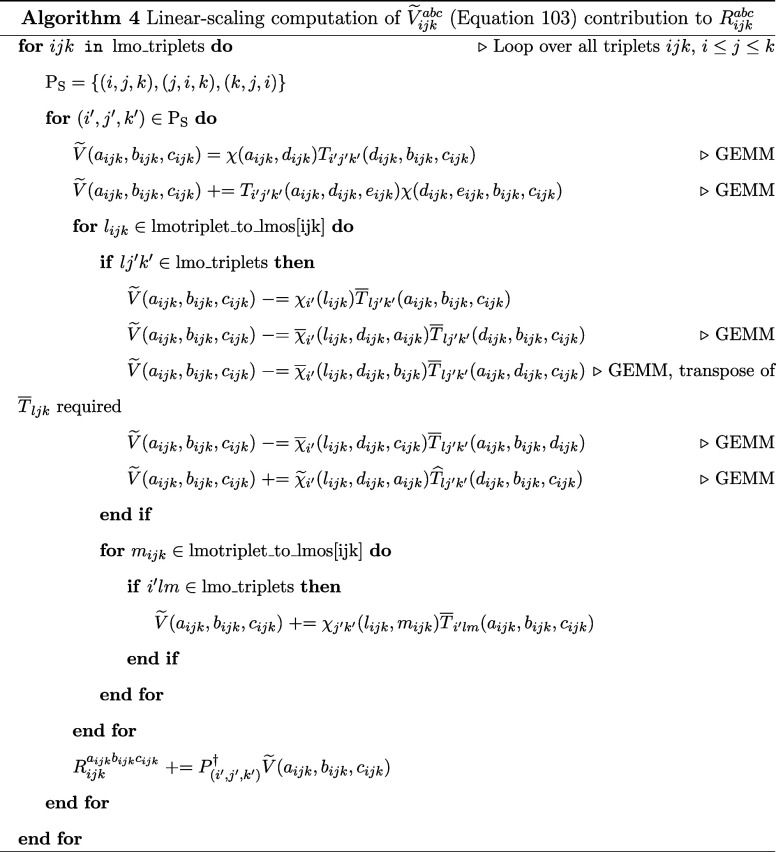


The strong and weak pair doubles residuals are updated with CCSDT triples amplitudes, and the semicanonical MP2 pair and dipole pair corrections, as well as PNO truncation error, are ported over from DLPNO–CCSD.^54^ The last term, or the triples truncation error, is ported from DLPNO–CCSD(T),^54^ and has the following form
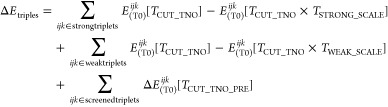
114

This is a measure of the error incurred for switching to a looser TNO tolerance after the initial DLPNO-(T0) energy computation, and includes the energy contribution of the screened triplets. The reader is referred to our previous work for more details on the various DLPNO-(T) components.^54^

## IV Implementation Details

For all correlated computations, the frozen-core approximation is used. The code can currently be found in a developmental plugin branch of the free and open-source Psi4 package.^56^ For tensor contractions, the Einsums library of Turney and Briggs is used.^118^ Currently, single node parallelization through OpenMP is utilized. Each presented algorithm is looped over LMO triplets, and triplets *ijk* are assigned to each thread based on how many TNOs are in its virtual space (*n*_tno_ijk_), for optimal load balancing and parallel efficiency. Unless otherwise stated, the preceding DLPNO–CCSD computation for DLPNO–CCSD(T)/T is evaluated using the TightPNO setting as defined in our prior work,^54^ with the exception that the *T*_DIAG_SCALE_ parameter has been revised to 3 × 10^–2^ instead of 10^–3^, to match the DLPNO–CCSD(T) implementation in ORCA.^55^

## V Results

### V.I Convergence of DLPNO–CCSDT

**Figure 1:**
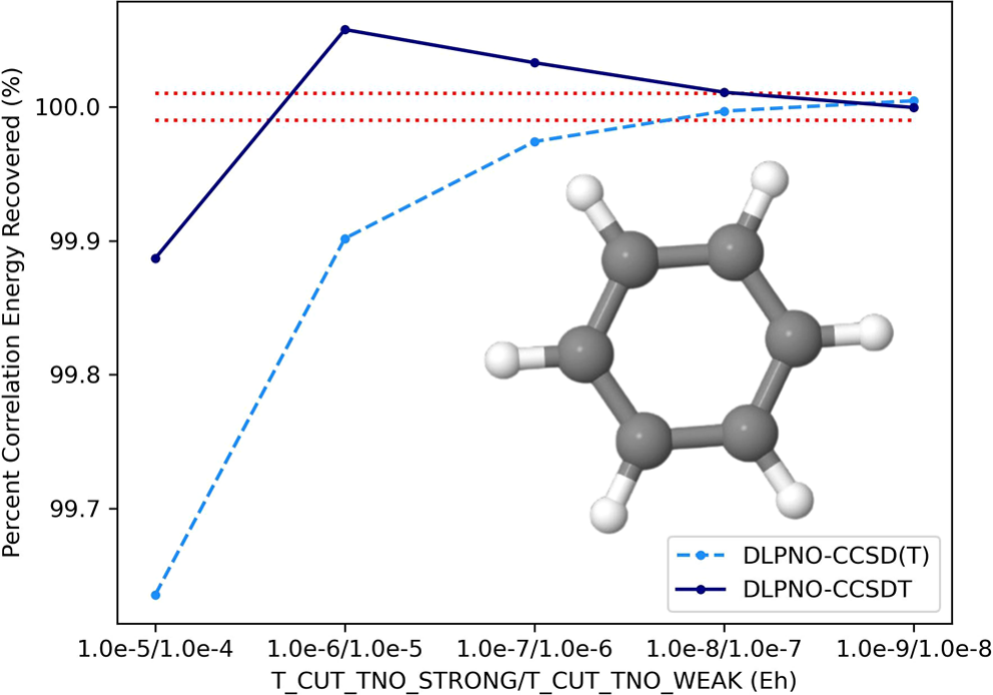
Convergence of the DLPNO–CCSD(T)/T correlation energy for benzene/cc-pVDZ with respect to the TNO tolerance for strong/weak triples. Dotted red lines represent target accuracy (less than 0.01% error in the correlation energy recovered).

**Figure 2:**
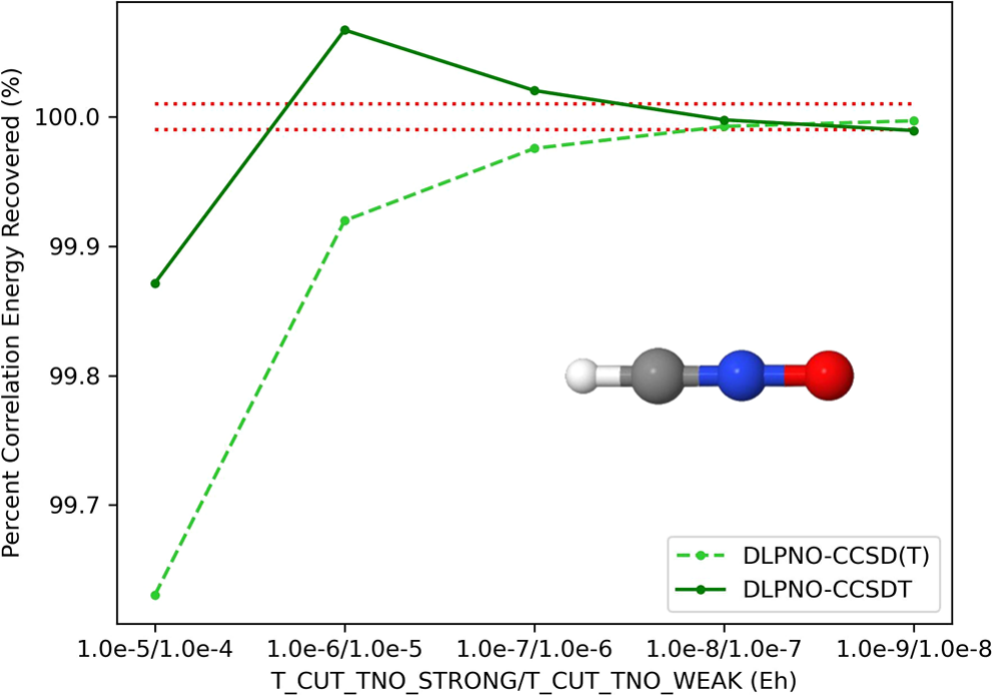
Convergence of the DLPNO–CCSD(T)/T correlation energy for HCNO/cc-pVTZ with respect to the TNO tolerance for strong/weak triples. Dotted red lines represent target accuracy (less than 0.01% error in the correlation energy recovered).

First, we evaluate the accuracy and convergence of our DLPNO–CCSDT algorithm on a benzene molecule (cc-pVDZ basis, 114 basis functions), as well as a linear fulminic acid (HCNO) structure (cc-pVTZ basis, 104 basis functions) in Figures 1 and 2, comparing it to the results of the preceding DLPNO–CCSD(T) computation. The geometry for the benzene molecule is provided in the Supporting Information, while the linear HCNO geometry is derived from the work of Allen et al., optimized at the AE-CCSDTQ(P)/CBS + MVD1 level of theory.^85^ The canonical CCSD(T) and CCSDT energies for benzene are computed through the MRCC software,^60^ while the reference energies for HCNO are derived from the work of Allen et al.^85^ For HCNO, a cc-pV5Z-RI basis set is used for the RI domain in our DLPNO computations to control for RI error. In Figures 1 and 2, the ratio of the DLPNO–CCSDT correlation energy with respect to the canonical CCSDT energy is computed as a function of the TNO tolerances of the strong and weak triplets (see Section II.III.V), and then compared to the percent recovery of the canonical CCSD(T) energy in the preceding DLPNO–CCSD(T) computation. Riplinger et al., for chemical accuracy, a good target is the recovery of at least 99.9% correlation energy.^47^ However, applications of CCSDT require an order of magnitude higher accuracy to reach kJ mol^–1^ accuracy. Therefore, a good standard for applications of DLPNO–CCSDT is to recover no less than 99.99% and no more than 100.01% of the canonical CCSDT correlation energy. The dotted red lines in Figures 1 and 2 represent our desired accuracy. In both cases, the target accuracy of CCSDT is achieved at a strong pair/weak pair TNO tolerance of 10^–8^/10^–7^, which happen to be the default settings for DLPNO–CCSD(T). Therefore, unless otherwise stated, all DLPNO–CCSDT computations reported in subsequent sections will be computed using these parameters. It is interesting to note the difference in convergence behavior between DLPNO–CCSD(T) and DLPNO–CCSDT, with the former monotonically increasing toward convergence, while the latter showing some oscillatory behavior at lower TNO tolerances before monotonically decreasing toward convergence at tighter tolerances. This is purely an artifact of the triples rank correction (eq 114) used in DLPNO–CCSDT computed at the DLPNO-(T0) level, which overestimates the magnitude of the correction at lower TNO tolerances (the TNO correction at the DLPNO-(T0) level is computed with T_CUT_TNO at 0.1 times T_CUT_TNO_STRONG). Without this correction, the DLPNO–CCSDT energy converges monotonically toward convergence, similar to DLPNO-(T), though a tighter tolerance is needed to reach 99.99% of the correlation energy (details included in SI). Using the triples rank correction, the target accuracy is reached for DLPNO–CCSD(T) and DLPNO–CCSDT at the same, looser set of parameters.

### V.II Pericyclic Reactions

**Figure 3:**
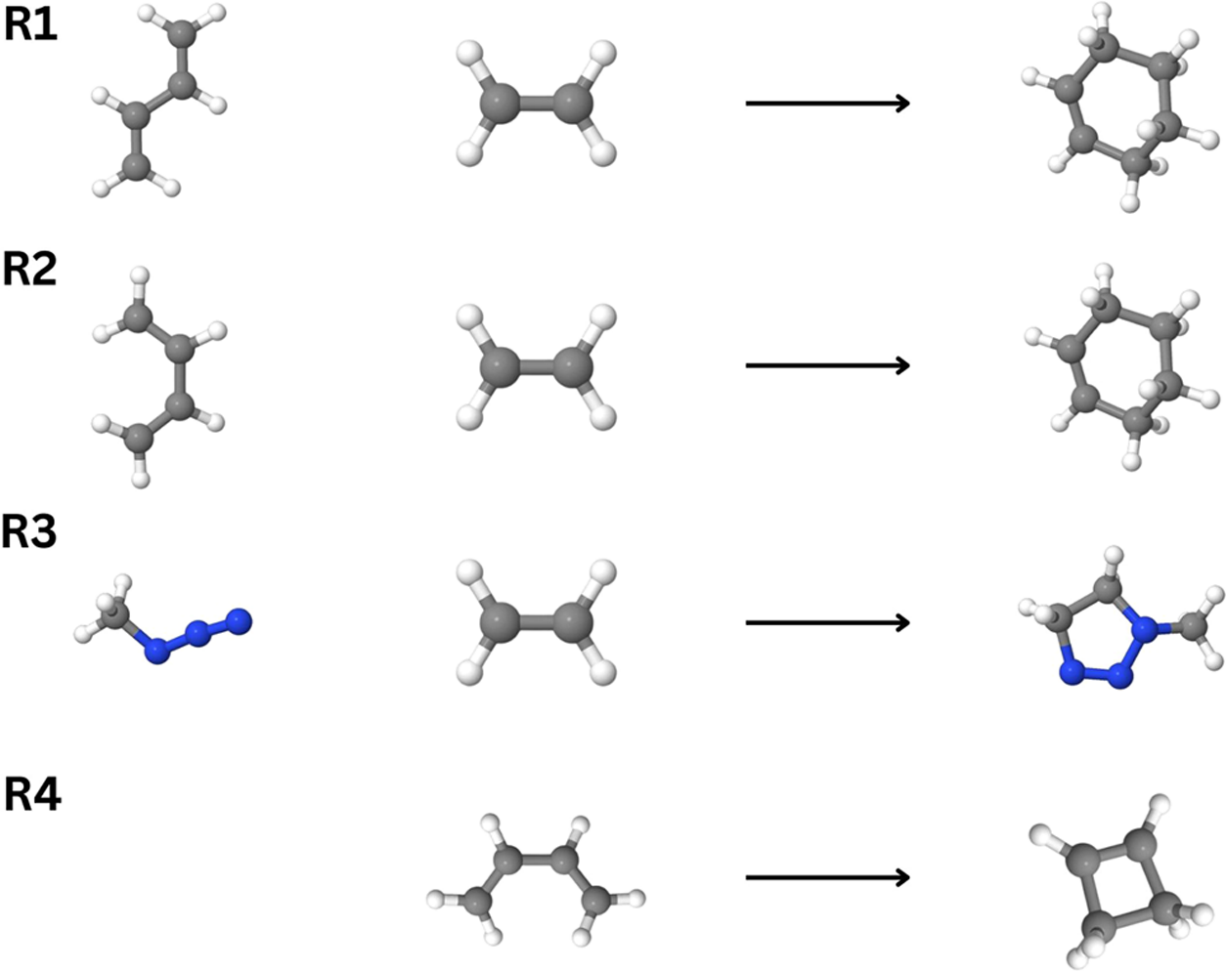
Representation of the geometries for the pericyclic reactions referenced in Table 2.

**Figure 4:**
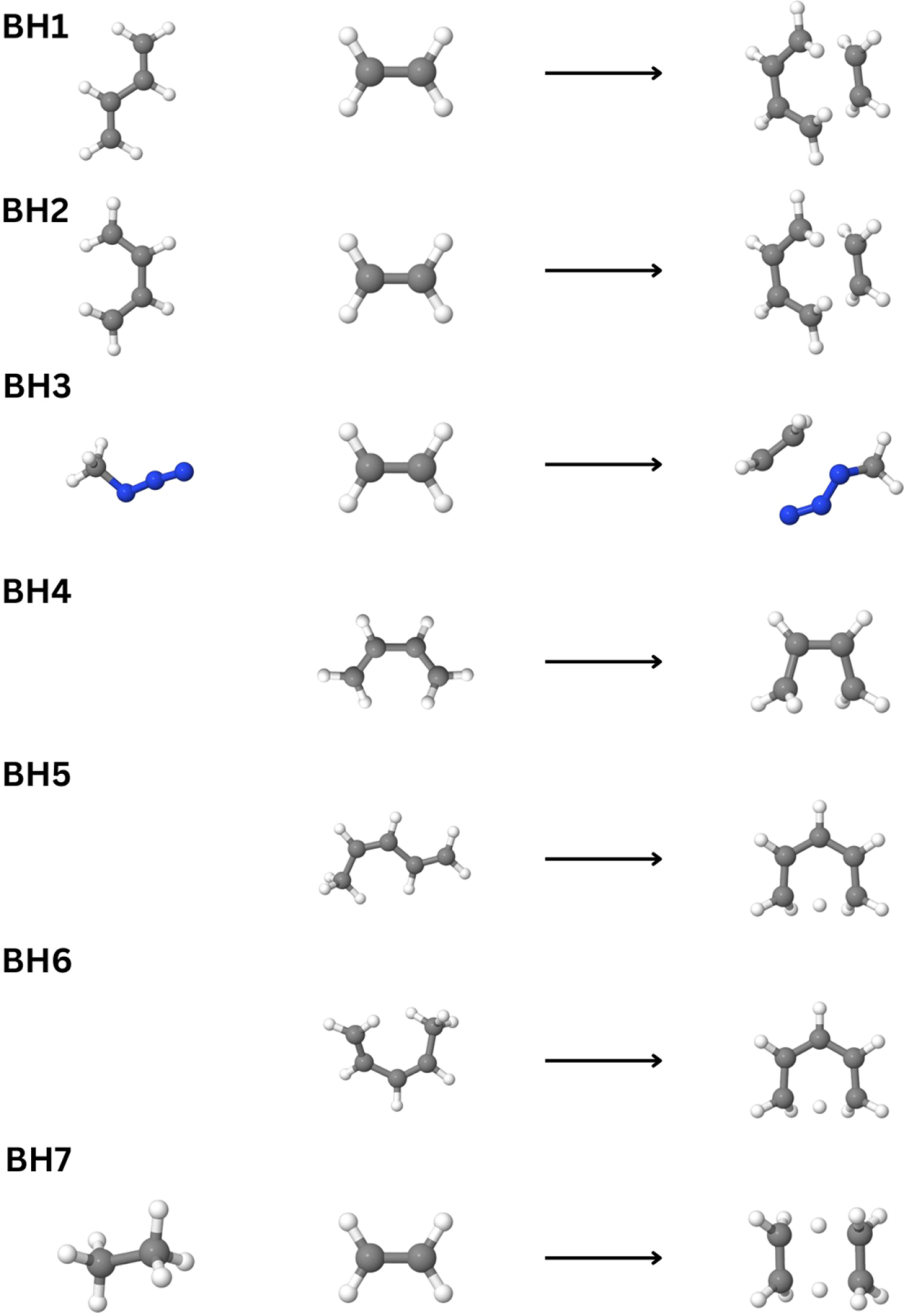
Representation of the geometries of the pericyclic barrier heights referenced in Table 3.

Next, we assess the accuracy of our DLPNO–CCSDT algorithm relative to the canonical CCSDT on a set of pericyclic reactions consisting of Diels–Alder, 1,3-dipolar cycloaddition, electrocyclic rearrangement, sigmatropic rearrangement, and double group transfer mechanisms. The reference geometries, optimized at the CCSD(T)/cc-pVTZ level of theory and energies (CCSDT/cc-pVDZ) are derived from the work of Vermeeren et al.^86^ Results for reaction energies are presented in Table 2, while barrier heights are presented in Table 3. For clarity, three-dimensional renderings of the reactant and product geometries are presented in Figures 3 and 4. The results of the DLPNO–CCSDT computation are compared with the canonical values (in kcal mol^–1^), with the errors presented in parentheses. For completeness, the difference between CCSDT and CCSD(T) values, denoted as δ_CCSDT_^CCSD(T)^, is reported, with the analogous DLPNO–CCSDT and DLPNO–CCSD(T) difference denoted δ_DLPNO–CCSDT_^DLPNO–CCSD(T)^. The computations are run at a TightPNO convergence for the preceding DLPNO–CCSD(T) computation and the default parameters for *T*_CUT_TNO_ (10^–9^), *T*_STRONG_SCALE_ (10), and *T*_WEAK_SCALE_ (100). The results show strong agreement between the CCSDT and DLPNO–CCSDT computed reaction enthalpies and barrier heights, with the highest error being 0.12 kcal mol^–1^ (around 0.5 kJ mol^–1^). It is an especially encouraging observation that the magnitude of the error between DLPNO–CCSDT and canonical CCSDT is invariant to the magnitude of δ_CCSDT_^CCSD(T)^. We do, however, strongly caution the use of taking the difference between DLPNO–CCSDT and DLPNO–CCSD(T) energies to estimate δ_CCSDT_^CCSD(T)^. Though the errors between δ_CCSDT_^CCSD(T)^ and δ_DLPNO–CCSDT_^DLPNO–CCSD(T)^ remain within 1 kJ mol^–1^ (0.24 kcal mol^–1^), we find that there is unfavorable error cancellation that occurs in most cases between DLPNO–CCSDT and DLPNO–CCSD(T). This is because the DLPNO-(T) energy is explicitly a function of the triples amplitude, while the DLPNO–CCSDT energy is only a functional of converged DLPNO–CCSDT singles and doubles amplitudes. This leads to a rank mismatch that yields unpredictable error cancellation. These issues are mitigated in cases of unimolecular reactions, denoted with (*) in Tables 2 and 3. In these cases, δ_DLPNO–CCSDT_^DLPNO–CCSD(T)^ approximates δ_CCSDT_^CCSD(T)^ quite accurately due to the lack of the rank error induced by the differences in system size between reagents and products. This is congruent with the observation of Gray and Herbert,^119^ that rank corrections such as the counterpoise (CP) correction are helpful when computing energy differences involving systems of different sizes, when used in the context of DLPNO. We recommend that for approximating δ_CCSDT_^CCSD(T)^ in focal point approaches, that the difference between DLPNO–CCSDT and canonical CCSD(T) should be used rather than the difference between DLPNO–CCSDT and DLPNO–CCSD(T).

**Table 2:** Pericyclic Reaction Energies for DLPNO–CCSDT Compared to Canonical CCSDT, along with the Relevant Energy Differences, Canonical Values Derived from the Work of Vermeeren et al.^86^^,^^a^

reaction	Δ*E*_CCSDT_	Δ*E*_DLPNO–CCSDT_	δ_CCSDT_^CCSD(T)^	δ_DLPNO–CCSDT_^DLPNO–CCSD(T)^
*trans* Diels–Alder reaction (R1)	–45.65	–45.77 (−0.12)	+0.37	+0.15 (−0.22)
Diels–Alder reaction (R2)	–48.59	–48.71 (−0.12)	+0.35	+0.13 (−0.22)
1,3-dipolar cycloaddition (R3)	–28.22	–28.20 (+0.02)	+0.11	–0.11 (−0.22)
electrocyclic rearrangement (R4)*	+8.37	+8.34 (−0.03)	+0.19	+0.16 (−0.03)

a Errors with respect to canonical values are in parentheses, and reactions denoted * represent a unimolecular reaction. All reported energies are in kcal mol^–1^ and computations performed using the cc-pVDZ basis.

**Table 3:** Pericyclic Barrier Heights for DLPNO–CCSDT Compared to Canonical CCSDT, along with the Relevant Energy Differences, Canonical Values Derived from the Work of Vermeeren et al.^86^^,^^a^

reaction	Δ*E*_CCSDT_	Δ*E*_DLPNO–CCSDT_	δ_CCSDT_^CCSD(T)^	δ_DLPNO–CCSDT_^DLPNO–CCSD(T)^
*trans* Diels–Alder reaction (BH1)	+24.54	+24.58 (+0.04)	+0.48	+0.32 (−0.16)
Diels–Alder reaction (BH2)	+21.61	+21.64 (+0.03)	+0.46	+0.30 (−0.16)
1,3-dipolar cycloaddition (BH3)	+18.59	+18.65 (+0.06)	+0.70	+0.51 (−0.19)
electrocyclic rearrangement (BH4)*	+43.64	+43.69 (+0.05)	+0.16	+0.18 (+0.02)
(*E*)-sigmatropic rearrangement (BH5)*	+40.00	+40.07 (+0.07)	+0.31	+0.32 (+0.01)
(*Z*)-sigmatropic rearrangement (BH6)*	+37.12	+37.20 (+0.08)	+0.27	+0.29 (+0.02)
double group transfer (BH7)	+51.35	+51.33 (−0.02)	+0.30	+0.22 (−0.08)

a Errors with respect to canonical values are in parentheses, and reactions denoted * represent a unimolecular reaction. All reported energies are in kcal mol^–1^ and computations performed using the cc-pVDZ basis.

### V.III Timings

**Figure 5:**
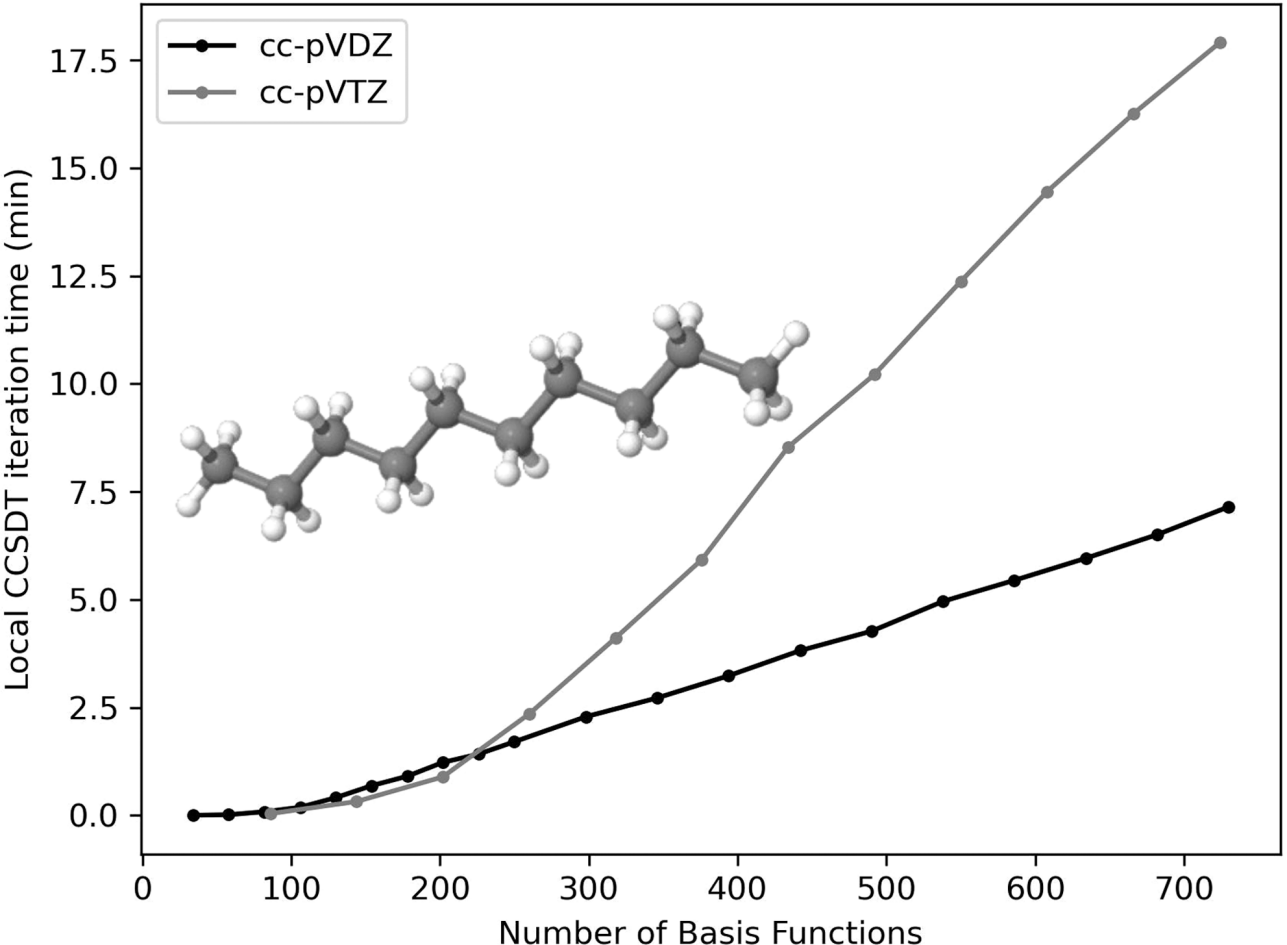
Linear alkane wall time per iteration for DLPNO–CCSDT. System sizes range from 1–30 carbons for the cc-pVDZ basis set, and 1–12 carbons for the cc-pVTZ basis set.

**Figure 6:**
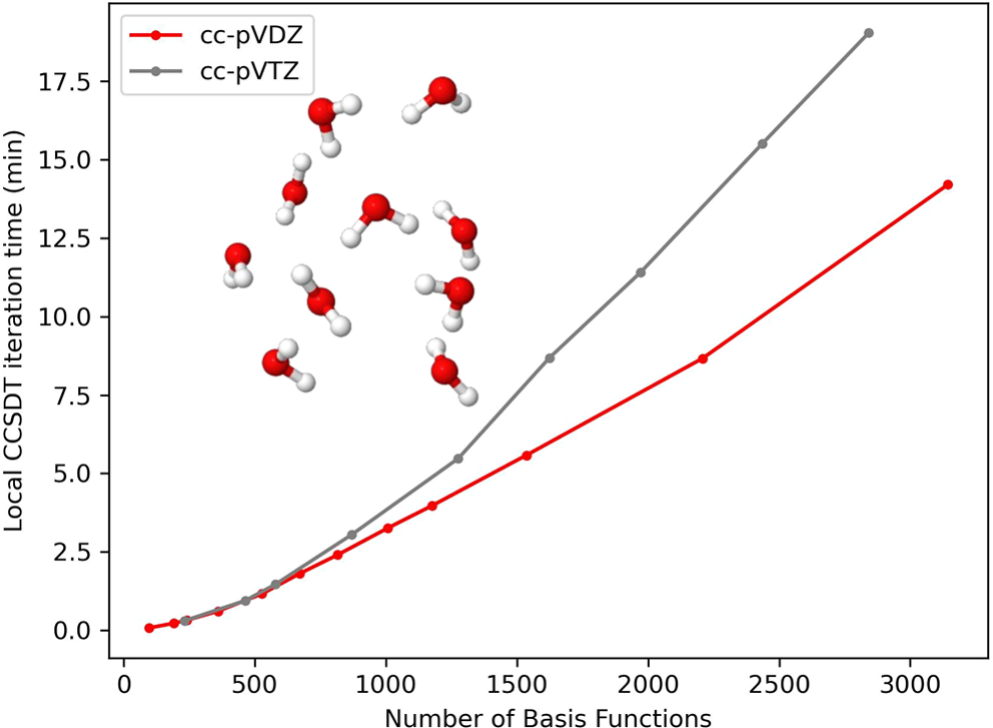
Water cluster wall time per iteration for DLPNO–CCSDT. System sizes range from 4–131 waters in cc-pVDZ, and 4–49 waters in cc-pVTZ.

Finally, to highlight the capabilities of our code, we performed timings on a growing series of linear alkanes as well as water clusters (geometries available in the Supporting Information), depicted in Figures 5 and 6. Results are presented using both the cc-pVDZ and cc-pVTZ basis sets, with the per-iteration DLPNO–CCSDT wall time (in minutes) evaluated with respect to the number of basis functions. For the alkanes, system sizes range from 1 to 30 carbons for cc-pVDZ (34 to 730 basis functions) and 1 to 12 carbons for cc-pVTZ (86 to 724 basis functions). For the water clusters, the system sizes range from 4 to 131 water molecules using cc-pVDZ (96 to 3144 basis functions) and 4 to 49 waters using cc-pVTZ (232 to 2842 basis functions). In these computations, the same parameters are used as the pericyclic reactions. As shown in Figures 5 and 6, the onset of linear scaling starts to occur in the range of these system sizes. To measure this, we performed a log–log regression of the per-iteration wall time with the number of basis functions, and fit the data to the function *t* = *a*·*N*^*b*^, where *t* is the run-time, *a* is a prefactor, *N* is the number of basis functions, and determined *b* (the observed scaling). For the linear alkanes, the observed scaling is around  for 12–30 carbon atoms in cc-pVDZ (*R*^2^ = 99.92%) and  for 10–12 carbons in cc-pVTZ (*R*^2^ = 99.90%). For the water clusters ranging from 34 to 131 molecules in the cc-pVDZ basis set, the observed scaling is , and  for 34–49 waters in the cc-pVTZ basis set. The *R*^2^ values are 99.94% across both basis sets. This gives evidence to support that our algorithm is asymptotically linear-scaling, given sufficiently large systems. All timings were performed using 48 CPU cores of an AMD EPYC 9274F (Genoa) with up to 2500 GB of RAM with a base clock of 4.05 GHz and a max clock of 4.3 GHz.

## VI Conclusions

In this work, we presented a novel domain-based local pair natural orbital CCSDT algorithm. We have shown it sufficiently accurate for use in chemical applications, yielding relative energy errors on the order of 0.5 kJ mol^–1^ or less, given sufficiently tight parameters. This algorithm is also convergent with canonical CCSDT. In addition, the asymptotic linear scaling of the algorithm allows it to be applied to much larger systems than previously studied with canonical CCSDT algorithms, as we present computations on systems with as many as 3144 basis functions. With this new algorithm, the obvious next step is to develop an analogous local natural orbital based linear-scaling local (Q) algorithm.^78^ These developments bridge the gap between theory and experiment by offering higher quality theoretical predictions for much larger molecules. These algorithmic improvements, combined with advances in computing hardware and hardware accelerators like GPUs,^22,23,120−144^ stretch the limits of computational quantum chemistry.

## Data Availability

The data that supports the findings of this study are available with the article and its Supporting Information. The Psi4 source code for DLPNO–CCSD(T) is available at https://github.com/andyj10224/psi4/tree/dlpno_ccsd_t_weak_pairs, while the code for the plugin for DLPNO–CCSDT is available at https://github.com/andyj10224/dlpno_ccsdt_q, and the code for Einsums is at https://github.com/Einsums/Einsums.

## Supporting Information Available

Algorithms for the computation of intermediates used in computing *W̃*_*ijk*_^*abc*^ and *Ṽ*_*ijk*_^*abc*^; graphs showing the contribution of the triples rank correction (Δ*E*_triples_) (PDF)
Geometries for the linear alkanes and water clusters, detailed timings for linear alkanes and water clusters, computed absolute energies for pericyclic reaction tests, computed absolute energies for fulminic acid and benzene case, as well as studies geometry for benzene (ZIP)
