# Supplementary material for: Linear-Scaling Local Natural Orbital-Based Full Triples Treatment in Coupled-Cluster Theory
Source: J Chem Theory Comput. 2025 Feb 21;21(5):2386–401. doi: 10.1021/acs.jctc.4c01716 (PMC11912218; doi:10.1021/acs.jctc.4c01716)
Supplement: Supplementary file 1 — ct4c01716_si_001.pdf [file ct4c01716_si_001.pdf]

Supplemental File For: Linear-Scaling Local Natural Orbital-based Full Triples Treatment in Coupled-Cluster Theory

Andy Jiang, Henry F. Schaefer III, and Justin M. Turney*

Center for Computational Quantum Chemistry,

Department of Chemistry, University of Georgia,

Athens, GA 30602, United States of America

* justin.turney@uga.edu

SI. PSEUDOCODE FOR t_1-DRESSING OF DF-INTEGRALS

For clarity, when DF integrals are presented before, we are assuming that they have already been orthogonalized by the necessary Coulomb metric

$$(Q_{ijk}|r_{ijk}s_{ijk}) \equiv B^{Q_{ijk}}_{r_{ijk}s_{ijk}} \tag{S1}$$

Algorithm S1 Linear-scaling t_1-dressing of DF integrals

```
for ijk in lmo_triplets do                                  ▷ Loop over all signficant triplets ijk, i ≤ j ≤ k
    B̃_i(Q_ijk, a_ijk) = (Q_ijk|i a_ijk)                      ▷ t1-dressing of B^Q_ai integrals, repeat for j and k
    B̃_i(Q_ijk, a_ijk) −= (Q_ijk|i l_ijk) T̄(l_ijk, a_ijk)                                              ▷ GEMM
    B̃_i(Q_ijk, a_ijk) += (Q_ijk|a_ijk b_ijk) T̄(i, b_ijk)                                              ▷ GEMM
    B̃_i(Q_ijk, a_ijk) −= T̄(l_ijk, b_ijk)(Q_ijk|l_ijk b_ijk) T̄(i, b_ijk)                             ▷ GEMM (x2)
    B̃_i(Q_ijk, l_ijk) = (Q_ijk|i l_ijk)                      ▷ t1-dressing of B^Q_li integrals, repeat for j and k
    B̃_i(Q_ijk, l_ijk) += (Q_ijk|l_ijk a_ijk) T̄(i, a_ijk)                                              ▷ GEMM
    B̃(Q_ijk, a_ijk, b_ijk) = (Q_ijk|a_ijk, b_ijk)                               ▷ t1-dressing of B^Q_ab integrals
    B̃(Q_ijk, a_ijk, b_ijk) −= (Q_ijk|a_ijk l_ijk) T̄(l_ijk, b_ijk)                                     ▷ GEMM
end for
```

We also present the pseudocode for computation of all Fock matrix intermediates that would be used in later algorithms. For clarity,

$$\widetilde{F}(l_{ijk}, d_{ijk}) \equiv \widetilde{F}_{l_{ijk}d_{ijk}} \ , \tag{S2}$$

$$\widetilde{F}(a_{ijk}, d_{ijk}) \equiv \widetilde{F}_{a_{ijk}d_{ijk}} \ , \tag{S3}$$

$$\widetilde{F}_i(l_{ijk}) \equiv \widetilde{F}_{l_{ijk}i} \tag{S4}$$

Algorithm S2 Linear-scaling computation of all Fock matrix intermediate

for ijk `in` lmo_triplets **do** ▷ Loop over all significant triplets ijk, $i \le j \le k$

$\Gamma_{Q_{ijk}} = (Q_{ijk}|m_{ijk}e_{ijk})\overline{T}(m_{ijk}, e_{ijk})$ ▷ GEMV

$\overline{F}(l_{ijk}, d_{ijk}) = 2(Q_{ijk}|l_{ijk}d_{ijk})\Gamma_{Q_{ijk}}$ ▷ GEMV

$A(Q_{ijk}, l_{ijk}, m_{ijk}) = (Q_{ijk}|l_{ijk}e_{ijk})\overline{T}(m_{ijk}, e_{ijk})$ ▷ GEMM

$A^T(Q_{ijk}, m_{ijk}, l_{ijk}) = A(Q_{ijk}, l_{ijk}, m_{ijk})$ ▷ GEMM

$\overline{F}(l_{ijk}, d_{ijk}) \mathrel{-}= A^T(Q_{ijk}, m_{ijk}, l_{ijk})(Q_{ijk}|m_{ijk}d_{ijk})$

$\widetilde{F}(l_{ijk}, d_{ijk}) \equiv \overline{F}(l_{ijk}, d_{ijk})$ ▷ $\widetilde{F}_{l_{ijk}d_{ijk}}$

$\overline{F}(a_{ijk}, d_{ijk}) = \epsilon_{a_{ijk}}\delta_{a_{ijk},d_{ijk}}$ ▷ Fock matrix is diagonal in TNO domain

$\overline{F}(a_{ijk}, d_{ijk}) \mathrel{+}= 2(Q_{ijk}|a_{ijk}d_{ijk})\Gamma_{Q_{ijk}}$ ▷ GEMV

$C(Q_{ijk}, a_{ijk}, m_{ijk}) = (Q_{ijk}|a_{ijk}e_{ijk})\overline{T}(m_{ijk}, e_{ijk})$ ▷ GEMM

$C^T(Q_{ijk}, m_{ijk}, a_{ijk}) = C(Q_{ijk}, a_{ijk}, m_{ijk})$

$\overline{F}(a_{ijk}, d_{ijk}) \mathrel{-}= C^T(Q_{ijk}, m_{ijk}, a_{ijk})(Q_{ijk}|m_{ijk}d_{ijk})$ ▷ GEMM

$\widetilde{F}(a_{ijk}, d_{ijk}) = \overline{F}(a_{ijk}, d_{ijk}) - \overline{T}(m_{ijk}, a_{ijk})\overline{F}(m_{ijk}, d_{ijk})$ ▷ GEMM, $\widetilde{F}_{a_{ijk}d_{ijk}}$

$\overline{F}_i(l_{ijk}) = F_{l_{ijk}i}$ ▷ Repeat for j and k

$\overline{F}_i(l_{ijk}) \mathrel{+}= 2(Q_{ijk}|l_{ijk}i)\Gamma_{Q_{ijk}}$ ▷ GEMV

$D(Q_{ijk}, a_{ijk}) = (Q_{ijk}|m_{ijk}i)\overline{T}(m_{ijk}, a_{ijk})$ ▷ GEMM

$\overline{F}_i(l_{ijk}) \mathrel{-}= (Q_{ijk}|a_{ijk}l_{ijk})D(Q_{ijk}, a_{ijk})$ ▷ GEMV

$\widetilde{F}_i(l_{ijk}) = \overline{F}_i(l_{ijk}) + \overline{T}(i, d_{ijk})\overline{F}(l_{ijk}, d_{ijk})$ ▷ GEMM, $\widetilde{F}_{l_{ijk}i}$, repeat for j and k

end for

SII. PSEUDOCODE FOR W INTERMEDIATES

Using the same notation as defined in the manuscript,

$$\rho_k(d_{ijk}, b_{ijk}, c_{ijk}) \equiv \rho^{b_{ijk}d_{ijk}}_{c_{ijk}k} , \tag{S5}$$

$$\rho_{jk}(l_{ijk}, c_{ijk}) \equiv \rho^{l_{ijk}j}_{c_{ijk}k} . \tag{S6}$$

In addition,

$$\overline{U}_{il}(l_{ijk}, a_{ijk}, b_{ijk}) \equiv 2\overline{T}_{il_{ijk}}(a_{ijk}, b_{ijk}) - \overline{T}_{il_{ijk}}(b_{ijk}, a_{ijk}) \ , \tag{S7}$$

$$\overline{U}_{li}(l_{ijk}, a_{ijk}, b_{ijk}) \equiv 2\overline{T}_{l_{ijk}i}(a_{ijk}, b_{ijk}) - \overline{T}_{l_{ijk}i}(b_{ijk}, a_{ijk}) \ , \tag{S8}$$

$$\overline{U}(l_{ijk}, m_{ijk}, a_{ijk}, b_{ijk}) \equiv 2\overline{T}_{l_{ijk}m_{ijk}}(a_{ijk}, b_{ijk}) - \overline{T}_{l_{ijk}m_{ijk}}(b_{ijk}, a_{ijk}) \ . \tag{S9}$$

Algorithm S3 Linear-scaling computation of $\rho_{c_{ijk}k}^{b_{ijk}d_{ijk}}$ intermediate

for ijk `in` lmo_triplets **do** ▷ Loop over all significant triplets ijk, $i \le j \le k$

  $\widetilde{g}(d_{ijk}, b_{ijk}, l_{ijk}, e_{ijk}) = \widetilde{B}(Q_{ijk}, d_{ijk}, b_{ijk})(Q_{ijk}|l_{ijk}e_{ijk})$ ▷ GEMM

  $\widetilde{g}^T(e_{ijk}, b_{ijk}, l_{ijk}, d_{ijk}) = \widetilde{g}(d_{ijk}, b_{ijk}, l_{ijk}, e_{ijk})$

  for $i' \in \{i, j, k\}$ **do**

    $\rho_{i'}(d_{ijk}, b_{ijk}, c_{ijk}) = \widetilde{B}(Q_{ijk}, d_{ijk}, b_{ijk})\widetilde{B}_{i'}(Q_{ijk}, c_{ijk})$ ▷ GEMM

    $\rho_{i'}(d_{ijk}, b_{ijk}, c_{ijk}) -= \widetilde{F}(l_{ijk}, d_{ijk})\overline{T}_{li'}(l_{ijk}, b_{ijk}, c_{ijk})$

    $g_{i'}(l_{ijk}, m_{ijk}, d_{ijk}) = \widetilde{B}_{i'}(Q_{ijk}, l_{ijk})(Q_{ijk}|m_{ijk}d_{ijk})$ ▷ GEMM

    $g_{i'}^T(m_{ijk}, l_{ijk}, d_{ijk}) = g_{i'}(l_{ijk}, m_{ijk}, d_{ijk})$

    $\rho_{i'}(d_{ijk}, b_{ijk}, c_{ijk}) += g_{i'}^T(m_{ijk}, l_{ijk}, d_{ijk})\overline{T}(m_{ijk}, l_{ijk}, b_{ijk}, c_{ijk})$ ▷ GEMM

    $\rho_{i'}(d_{ijk}, b_{ijk}, c_{ijk}) += \widetilde{g}(d_{ijk}, b_{ijk}, l_{ijk}, e_{ijk})\overline{U}_{li'}(l_{ijk}, e_{ijk}, c_{ijk})$ ▷ GEMM

    $\rho_{i'}(d_{ijk}, b_{ijk}, c_{ijk}) -= \widetilde{g}^T(d_{ijk}, b_{ijk}, l_{ijk}, e_{ijk})\overline{T}_{li'}(l_{ijk}, e_{ijk}, b_{ijk})$ ▷ GEMM

    $\overline{\rho}(d_{ijk}, c_{ijk}, b_{ijk}) = \widetilde{g}^T(d_{ijk}, c_{ijk}, l_{ijk}, e_{ijk})\overline{T}_{il'}(l_{ijk}, e_{ijk}, b_{ijk})$ ▷ GEMM

    $\rho_{i'}(d_{ijk}, b_{ijk}, c_{ijk}) -= \overline{\rho}(d_{ijk}, c_{ijk}, b_{ijk})$

    for $m_{ijk} \in$ lmotriplet_to_lmos[ijk] **do**

      for $l_{ijk} \in$ lmotriplet_to_lmos[ijk] **do**

        if $mli' \in$ lmo_triplets **then**

          $g_{ml}(e_{ijk}, d_{ijk}) = (m_{ijk}e_{ijk}|l_{ijk}d_{ijk})$

          $\widetilde{T}_{mli}(e_{ijk}, b_{ijk}, c_{ijk}) = 2\overline{t}_{mli}^{e_{ijk}b_{ijk}c_{ijk}} - \overline{t}_{mli}^{c_{ijk}b_{ijk}e_{ijk}} - \overline{t}_{mli}^{b_{ijk}e_{ijk}c_{ijk}}$

          $\rho_{i'}(d_{ijk}, b_{ijk}, c_{ijk}) -= g_{ml}(e_{ijk}, d_{ijk})\widetilde{T}_{mli}(e_{ijk}, b_{ijk}, c_{ijk})$ ▷ GEMM

        end if

      end for

    end for

  end for

end for

Algorithm S4 Linear-scaling computation of $\rho^{l_{ijk}j}_{c_{ijk}k}$ intermediate

for ijk `in` lmo_triplets **do** ▷ Loop over all significant triplets ijk, $i \leq j \leq k$

$\widetilde{g}(d_{ijk}, b_{ijk}, l_{ijk}, e_{ijk}) = \widetilde{B}(Q_{ijk}, d_{ijk}, b_{ijk})(Q_{ijk}|l_{ijk}e_{ijk})$ ▷ GEMM, defined in previous algorithm

$\widetilde{g}^*(l_{ijk}, c_{ijk}, e_{ijk}, d_{ijk}) = \widetilde{g}(d_{ijk}, c_{ijk}, l_{ijk}, e_{ijk})$

$\mathrm{P_L} = \{(i,j,k),(i,k,j),(j,i,k),(j,k,i),(k,i,j),(k,j,i)\}$

for $(i', j', k') \in \mathrm{P_L}$ **do**

$\rho_{j'k'}(l_{ijk}, c_{ijk}) = \widetilde{B}_{j'}(Q_{ijk}, l_{ijk})\widetilde{B}_{k'}(Q_{ijk}, c_{ijk})$ ▷ GEMM

$\widetilde{g}_{j'}(m_{ijk}, d_{ijk}, l_{ijk}) = (Q_{ijk}|m_{ijk}d_{ijk})\widetilde{B}_{j'}(Q_{ijk}, l_{ijk})$ ▷ GEMM

$\rho_{j'k'}(l_{ijk}, c_{ijk}) \mathrel{+}= \widetilde{g}_{j'}(m_{ijk}, d_{ijk}, l_{ijk})\overline{U}_{mk'}(m_{ijk}, d_{ijk}, c_{ijk})$ ▷ GEMM

$\widetilde{g}^T_{j'}(l_{ijk}, m_{ijk}, d_{ijk}) = \widetilde{g}_{j'}(l_{ijk}, d_{ijk}, m_{ijk})$

$\rho_{j'k'}(l_{ijk}, c_{ijk}) \mathrel{-}= \widetilde{g}^T_{j'}(l_{ijk}, m_{ijk}, d_{ijk})\overline{T}_{mk'}(m_{ijk}, d_{ijk}, c_{ijk})$ ▷ GEMM

$\widetilde{g}_{k'}(l_{ijk}, d_{ijk}, m_{ijk}) = (Q_{ijk}|l_{ijk}d_{ijk})\widetilde{B}_{k'}(Q_{ijk}, m_{ijk})$ ▷ GEMM

$\widetilde{g}^T_{k'}(l_{ijk}, m_{ijk}, d_{ijk}) = \widetilde{g}_{k'}(l_{ijk}, d_{ijk}, m_{ijk})$

$\rho_{j'k'}(l_{ijk}, c_{ijk}) \mathrel{-}= \widetilde{g}^T_{k'}(l_{ijk}, m_{ijk}, d_{ijk})\overline{T}_{j'm}(m_{ijk}, d_{ijk}, c_{ijk})$ ▷ GEMM

$\rho_{j'k'}(l_{ijk}, c_{ijk}) \mathrel{-}= \widetilde{g}^*(l_{ijk}, c_{ijk}, e_{ijk}, d_{ijk})\overline{T}_{j'k'}(e_{ijk}, d_{ijk})$ ▷ GEMM

for $m_{ijk} \in$ lmotriplet_to_lmos[ijk] **do**

if $mj'k' \in$ lmo_triplets **then**

$g_{ml}(e_{ijk}, d_{ijk}) = (m_{ijk}e_{ijk}|l_{ijk}d_{ijk})$

$\rho_{j'k'}(l_{ijk}, c_{ijk}) \mathrel{+}= g_{ml}(e_{ijk}, d_{ijk})\widehat{T}_{mj'k'}(e_{ijk}, d_{ijk}, c_{ijk})$ ▷ GEMM

end if

end for

end for

end for

SIII. PSEUDOCODE FOR V INTERMEDIATES

Using the same notation as defined in the manuscript,

$$\chi(a_{ijk}, d_{ijk}) \equiv \chi_{a_{ijk}d_{ijk}} \ , \tag{S10}$$

$$\chi(d_{ijk}, e_{ijk}, b_{ijk}, c_{ijk}) \equiv \chi_{b_{ijk}d_{ijk}}^{c_{ijk}e_{ijk}} \ , \tag{S11}$$

$$\chi_i(l_{ijk}) \equiv \chi_{l_{ijk}i} \ , \tag{S12}$$

$$\overline{\chi}_i(l_{ijk}, d_{ijk}, a_{ijk}) \equiv \chi_{a_{ijk}d_{ijk}}^{l_{ijk}i} \ , \tag{S13}$$

$$\widetilde{\chi}_i(l_{ijk}, d_{ijk}, a_{ijk}) \equiv \chi_{a_{ijk}i}^{l_{ijk}d_{ijk}} \ , \tag{S14}$$

$$\chi_{jk}(l_{ijk}, m_{ijk}) \equiv \chi_{l_{ijk}j}^{m_{ijk}k} \ , \tag{S15}$$

$$\widehat{T}_{ljk}(d_{ijk}, b_{ijk}, c_{ijk}) \equiv (2\overline{t}_{ljk}^{d_{ijk}b_{ijk}c_{ijk}} - \overline{t}_{ljk}^{c_{ijk}b_{ijk}d_{ijk}} - \overline{t}_{ljk}^{b_{ijk}d_{ijk}c_{ijk}}) \ . \tag{S16}$$

Algorithm S5 Linear-scaling computation of $\chi_{a_{ijk}d_{ijk}}$ intermediate

for ijk `in` lmo_triplets **do** ▷ Loop over all significant triplets ijk, $i \leq j \leq k$

  $\chi(a_{ijk}, d_{ijk}) = \widetilde{F}(a_{ijk}, d_{ijk})$

  $g(m_{ijk}, l_{ijk}, e_{ijk}, d_{ijk}) = (m_{ijk}e_{ijk}|l_{ijk}d_{ijk})$

  $\chi(a_{ijk}, d_{ijk}) \mathrel{-}= \overline{U}(m_{ijk}, l_{ijk}, e_{ijk}, a_{ijk})g(m_{ijk}, l_{ijk}, e_{ijk}, d_{ijk})$ ▷ GEMM

end for

Algorithm S6 Linear-scaling computation of $\chi_{b_{ijk}d_{ijk}}^{c_{ijk}e_{ijk}}$ intermediate

for ijk `in` lmo_triplets **do** ▷ Loop over all significant triplets ijk, $i \leq j \leq k$

  $\overline{\chi}(d_{ijk}, b_{ijk}, e_{ijk}, c_{ijk}) = \widetilde{B}(Q_{ijk}, d_{ijk}, b_{ijk})\widetilde{B}(Q_{ijk}, e_{ijk}, c_{ijk})$ ▷ GEMM

  $\chi(d_{ijk}, e_{ijk}, b_{ijk}, c_{ijk}) = \overline{\chi}(d_{ijk}, b_{ijk}, e_{ijk}, c_{ijk})$

  $g(l_{ijk}, m_{ijk}, d_{ijk}, e_{ijk}) = (l_{ijk}d_{ijk}|m_{ijk}e_{ijk})$

  $\chi(d_{ijk}, e_{ijk}, b_{ijk}, c_{ijk}) \mathrel{+}= g(l_{ijk}, m_{ijk}, d_{ijk}, e_{ijk})\overline{T}(l_{ijk}, m_{ijk}, b_{ijk}, c_{ijk})$ ▷ GEMM

end for

Algorithm S7 Linear-scaling computation of $\chi_{l_{ijk}i}$ intermediate

for ijk `in` lmo_triplets **do** ▷ Loop over all significant triplets ijk, $i \leq j \leq k$

  for $i' \in \{i, j, k\}$ **do**

    $\chi_{i'}(l_{ijk}) = \widetilde{F}_{i'}(l_{ijk})$

    $\widetilde{\chi}_{i'}(Q_{ijk}, d_{ijk}) = (Q_{ijk}|m_{ijk}e_{ijk})\overline{U}_{mi'}(m_{ijk}, e_{ijk}, d_{ijk})$ ▷ GEMM

    $\chi_{i'}(l_{ijk}) \mathrel{+}= (Q_{ijk}|d_{ijk}l_{ijk})\widetilde{\chi}_{i'}(Q_{ijk}, d_{ijk})$ ▷ GEMV

  end for

end for

Algorithm S8 Linear-scaling computation of $\chi^{l_{ijk}i}_{a_{ijk}d_{ijk}}$ intermediate

for ijk `in` lmo_triplets **do** ▷ Loop over all significant triplets ijk, $i \leq j \leq k$

  for $i' \in \{i, j, k\}$ **do**

    $\overline{\chi}_{i'}(l_{ijk}, d_{ijk}, a_{ijk}) = \widetilde{B}_{i'}(Q_{ijk}, l_{ijk})\widetilde{B}(Q_{ijk}, d_{ijk}, a_{ijk})$ ▷ GEMM

    $g^T(m_{ijk}, e_{ijk}, l_{ijk}, d_{ijk}) = (m_{ijk}d_{ijk}|l_{ijk}e_{ijk})$

    $\overline{\chi}_{i'}(l_{ijk}, d_{ijk}, a_{ijk}) \mathrel{-}= g^T(m_{ijk}, e_{ijk}, l_{ijk}, d_{ijk})\overline{T}_{i'm}(m_{ijk}, e_{ijk}, a_{ijk})$ ▷ GEMM

  end for

end for

Algorithm S9 Linear-scaling computation of $\chi^{l_{ijk}d_{ijk}}_{a_{ijk}i}$ intermediate

for ijk `in` lmo_triplets **do** ▷ Loop over all significant triplets ijk, $i \leq j \leq k$

  for $i' \in \{i, j, k\}$ **do**

    $\widetilde{\chi}_{i'}(l_{ijk}, d_{ijk}, a_{ijk}) = (Q_{ijk}|l_{ijk}d_{ijk})\widetilde{B}_{i'}(Q_{ijk}, a_{ijk})$ ▷ GEMM

    $g^T(l_{ijk}, d_{ijk}, m_{ijk}, e_{ijk}) = (l_{ijk}e_{ijk}|m_{ijk}d_{ijk})$

    $\widetilde{\chi}_{i'}(l_{ijk}, d_{ijk}, a_{ijk}) \mathrel{-}= g^T(l_{ijk}, d_{ijk}, m_{ijk}, e_{ijk})\overline{T}_{mi'}(m_{ijk}, e_{ijk}, a_{ijk})$ ▷ GEMM

    $\widetilde{\chi}_{i'}(l_{ijk}, d_{ijk}, a_{ijk}) \mathrel{+}= (l_{ijk}d_{ijk}|m_{ijk}e_{ijk})\overline{U}_{mi'}(m_{ijk}, e_{ijk}, a_{ijk})$ ▷ GEMM

  end for

end for

Algorithm S10 Linear-scaling computation of $\chi_{l_{ijk}j}^{m_{ijk}k}$ intermediate

for ijk `in` lmo_triplets **do** ▷ Loop over all significant triplets ijk, $i \le j \le k$

  $\mathrm{P_S} = \{(i,j,k),(j,i,k),(k,j,i)\}$

  for $(i',j',k') \in \mathrm{P_S}$ **do**

    $\chi_{j'k'}(l_{ijk}, m_{ijk}) = \widetilde{B}_{j'}(Q_{ijk}, l_{ijk})\widetilde{B}_{k'}(Q_{ijk}, m_{ijk})$ ▷ GEMM

    $\overline{\chi}_{j'k'}(Q_{ijk}, l_{ijk}, e_{ijk}) = (Q_{ijk}|l_{ijk}d_{ijk})\overline{T}_{j'k'}(d_{ijk}, e_{ijk})$ ▷ GEMM

    $\overline{\chi}^{T}_{j'k'}(Q_{ijk}, e_{ijk}, l_{ijk}) = \overline{\chi}_{j'k'}(Q_{ijk}, l_{ijk}, e_{ijk})$

    $\chi_{j'k'}(l_{ijk}, m_{ijk}) \mathrel{+}= \overline{\chi}^{T}_{j'k'}(Q_{ijk}, e_{ijk}, l_{ijk})(Q_{ijk}|e_{ijk}m_{ijk})$ ▷ GEMM

  end for

end for

SIV. EFFECT OF THE TRIPLES RANK CORRECTION ($\Delta E_{\mathrm{triples}}$)

In Section V A in the manuscript, we discussed the impact of the ($\Delta E_{\mathrm{triples}}$) correction on the total LCCSDT correlation energy, and how it allows for the recovery of the target 99.99% of the total CCSDT correlation energy using a looser triples natural orbitals (TNOs) tolerance. In this section, we explicitly show the effect of $\Delta E_{\mathrm{triples}}$ (Equation 114 in manuscript) for both benzene in cc-pVDZ and linear fulminic acid in cc-pVTZ. We note that the triples energy for DLPNO-(T0) is computed using a tighter TNO tolerance of 0.1 times T_CUT_TNO_STRONG or 0.01 times T_CUT_TNO_WEAK as shown in the figures below. For reader clarity, and as a reminder, the DLPNO-CCSD(T) values reflect the amount of correlation recovered with respect to canonical CCSD(T), and the CCSDT values reflect the amount of correlation recovered with respect to full CCSDT. In the figures below, we show that the triples rank correction, though it can lead to an over-correction at lower TNO tolerances, allows for the recovery of the target accuracy more efficently.

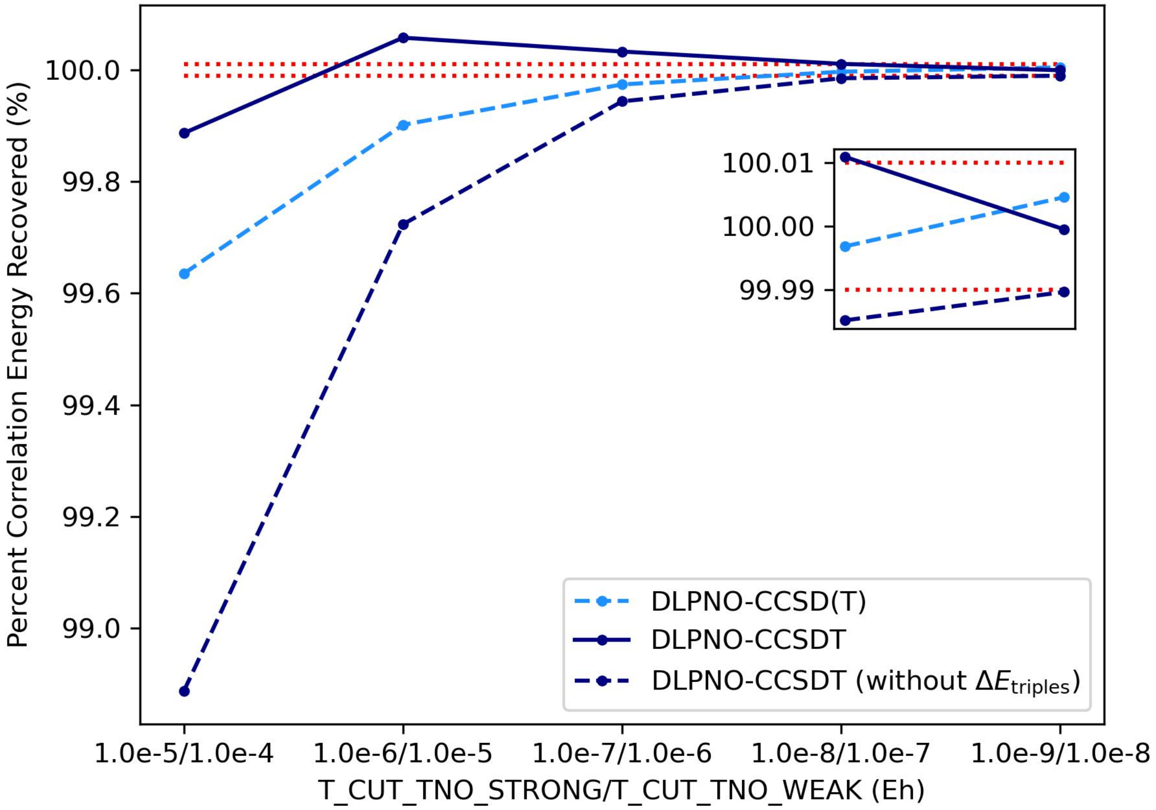

FIG. S1. The convergence of the DLPNO-CCSD(T)/T correlation energy for benzene/cc-pVDZ with respect to the TNO tolerance for strong/weak triples. The contribution of $\Delta E_{\text{triples}}$ is evaluated at each tolerance. The graph at $10^{-8}/10^{-7}$ and $10^{-9}/10^{-8}$ is zoomed in for clarity. Dotted red lines represent target accuracy (less than 0.01% error in the correlation energy recovered)

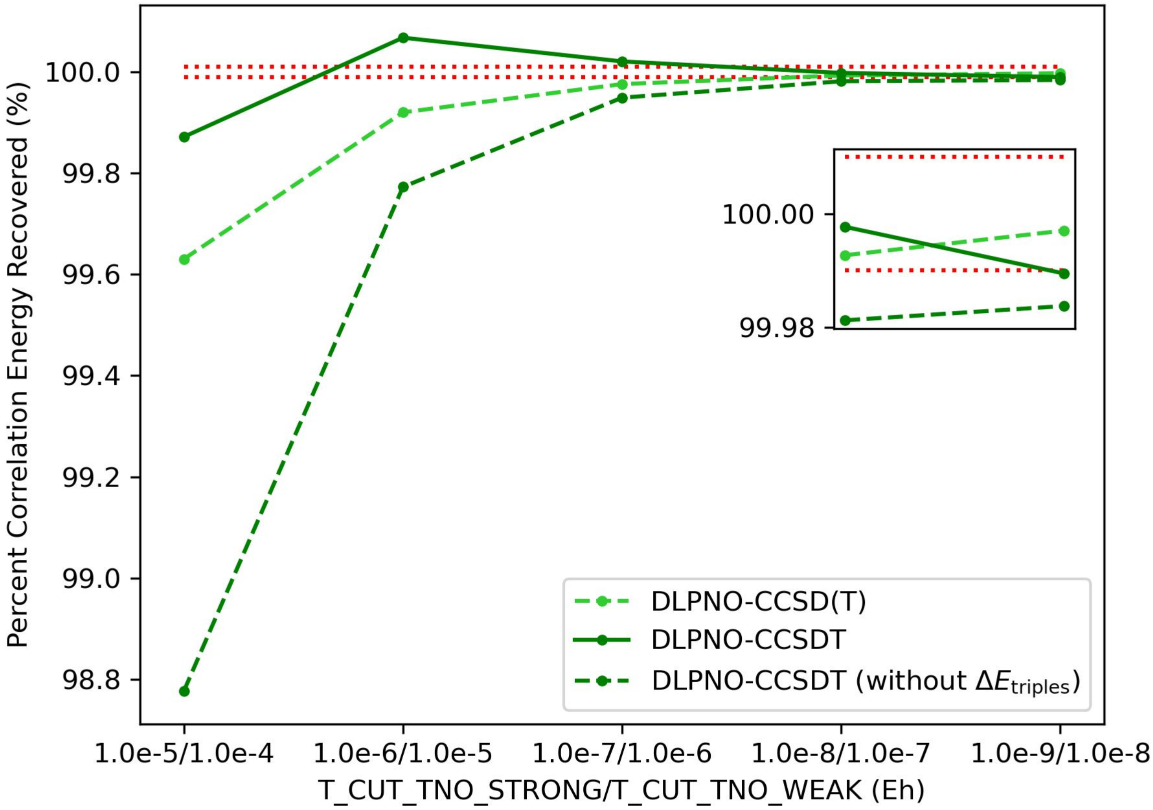

FIG. S2. The convergence of the DLPNO-CCSD(T)/T correlation energy for HCNO/cc-pVTZ with respect to the TNO tolerance for strong/weak triples. The contribution of $\Delta E_{\text{triples}}$ is evaluated at each tolerance. The graph at $10^{-8}/10^{-7}$ and $10^{-9}/10^{-8}$ is zoomed in for clarity. Dotted red lines represent target accuracy (less than 0.01% error in the correlation energy recovered.)
